# A New Journal

**Published:** 1882-02

**Authors:** 


### A NEW JOURNAL.

The first number of *The New England Journal of Dentistry and Allied Sciences*, published in Springfield, Mass., by the New England Journal Company, and edited by Associated Dentists, Chas. Mayr, with A.M.B.S., Scientific Editor, has just made its appearance. This is a monthy journal of thirty-two pages, filled with good and interesting matter. This is the first dental journal that has made its appearance in New England. It has been a matter of wonder often expressed that New England has not long ago produced a dental journal. It certainly was not because there were not men amply qualified for the work, for new England has had many of the most startling men of our profession. But, perhaps, they thought, as many have, that there has, all the while, been journa lsenough, or they may have preferred to speak through those already established. As to this there need be no criticism now: suffice it to say that they have made a good presentation in the first number. Long may it live and prosper, and be the means of doing good, is the wish of the REGISTER.

